# A Rodent Model of Exposure Therapy: The Use of Fear Extinction as a Therapeutic Intervention for PTSD

**DOI:** 10.3389/fnbeh.2019.00046

**Published:** 2019-03-11

**Authors:** Denisse Paredes, David A. Morilak

**Affiliations:** ^1^Department of Pharmacology and Center for Biomedical Neuroscience, University of Texas Health Science Center, San Antonio, San Antonio, TX, United States; ^2^South Texas Veterans Health Care System (STVHCS), San Antonio, TX, United States

**Keywords:** cognitive flexibility, coping, chronic unpredictable stress, infralimbic cortex, set shifting

## Abstract

The symptoms of post-traumatic stress disorder (PTSD) include cognitive impairment related to medial prefrontal cortical dysfunction. Indeed, a deficit of cognitive flexibility, i.e., an inability to modify previously learned thoughts and behaviors based on changes in the environment, may underlie many of the other symptoms of PTSD, such as changes in mood, hyper-arousal, intrusive thoughts, exaggerated and over-generalized fear, and avoidance behavior. Cognitive-behavioral therapies target the cognitive dysfunction observed in PTSD patients, training them to recalibrate stress-related perceptions, interpretations and responses. Preclinically, the extinction of conditioned fear bears resemblance to one form of cognitive therapy, exposure therapy, whereby an individual learns, through repeated exposure to a fear-provoking stimulus in a safe environment, that the stimulus no longer signals imminent threat, and their fear response is suppressed. In this review article, we highlight recent findings from our lab using fear extinction as a preclinical model of exposure therapy in rodents exposed to chronic unpredictable stress (CUS). We specifically focus on the therapeutic effects of extinction on stress-compromised set-shifting as a measure of cognitive flexibility, and active vs. passive coping behavior as a measure of avoidance. Finally, we discuss mechanisms involving activity and plasticity in the medial prefrontal cortex (mPFC) necessary for the therapeutic effects of extinction on cognitive flexibility and active coping.

## Introduction

Post-traumatic stress disorder (PTSD) is a debilitating illness that affects up to 8% of the general population in the United States (Kilpatrick et al., 2013), and as many as 20%–30% of combat veterans (Breslau, 2001). Development of PTSD symptoms is associated with experiencing or witnessing perceived life-threatening events, such as combat-related trauma, sexual abuse, and other uncontrollable and unpredictable events (Ozer et al., 2003). PTSD symptoms include fear generalization, intrusive re-experiencing of trauma, avoidance behaviors, cognitive impairments, and negative alterations in mood (DSM-V). PTSD has classically been conceptualized as a disorder of fear dysregulation, a traumatic event may subsequently cause an individual to generalize their fear of stimuli associated with the traumatic event to non-threatening stimuli, or to similar stimuli in non-threatening environments. Animal models of PTSD have historically focused on mimicking the exaggerated fear responses observed in the patient population (Foa et al., 1992, 2006). Preclinical PTSD research studies often utilize Pavlovian fear conditioning and extinction as dependent measures to investigate the neurobiology underlying the exacerbation of fear in PTSD (Milad and Quirk, 2012). The neurobiological circuitry involved in fear acquisition and **fear extinction** has been extensively studied and delineated [see reviews by Johnson et al. (2012) and VanElzakker et al. (2014)]. Using this approach, investigations aimed at developing strategies to improve PTSD symptoms have identified substances that can accelerate the rate of extinction learning (Milad and Quirk, 2012). However, while excessively strong conditioned fear is a central component of the illness, PTSD is a complex and chronic disorder, encompassing other symptom domains that reflect, for example, avoidance behavior and withdrawal, and disruptions of executive function and cognition. These other symptom domains may not appear at first glance to be directly related to aberrant fear memory. However, chronic PTSD and the repeated process of re-experiencing fearful memories, i.e., the constant retrieval and reactivation of conditioned fear, may in itself induce a state of chronic stress. This state of chronic **stress** could then secondarily impair the function of brain regions such as the prefrontal cortex (PFC; see, for example Jett et al., 2017), contributing to the development and maintenance of a broader array of symptoms than present initially, including disruptions of executive function and **cognitive flexibility** that are characteristic of PTSD.

KEY CONCEPT 1 **Fear extinction**

A decrease in conditioned fear responses (i.e., freezing) after repeated exposures of a non-reinforced conditioned stimulus (e.g., a tone).

KEY CONCEPT 2 **Stress**

Any threat, either real or perceived, to one’s health or well-being, that exceeds homeostatic regulatory capacity.

KEY CONCEPT 3 **Cognitive flexibility**

The ability to modify previously learned thoughts, behaviors or associations based on new information from the environment.

Indeed, PTSD patients exhibit hypoactivity of the ventromedial and dorsomedial PFC (Etkin and Wager, 2007). The medial PFC (mPFC) exerts a top-down inhibitory influence on the fear and anxiety elicited by amygdala activation (Koenigs and Grafman, 2009; Likhtik et al., 2014), so dysregulation of mPFC function could contribute directly to inappropriate regulation and disinhibition of amygdala activation, and the resulting fear and anxiety in PTSD (Goossens et al., 2007). In addition, patients with PTSD also exhibit impairments of other higher order cognitive processes and executive functions mediated in the PFC, such as set shifting, spatial working memory, and response inhibition (Olff et al., 2014). They perform poorly in tests of cognitive flexibility, such as the Wisconsin Card Sorting Test (Kanagaratnam and Asbjørnsen, 2007). The PFC is involved in executive function (Girotti et al., 2018). Thus, prefrontal hypoactivity may contribute to the cognitive dysfunction observed in PTSD patients.

KEY CONCEPT 4 **Coping**

Behavioral strategies mounted in response to a threatening stimulus or situation that serve to reduce or remove the threat, or to remove oneself from the threat.

In addition to deficits in cognitive flexibility, individuals with PTSD also often adopt passive **coping strategies**, associated with symptoms of avoidance and withdrawal (Olff et al., 2005). Passive coping strategies are associated with a greater neuroendocrine response to threat, and increase the likelihood that an individual will develop PTSD symptoms (Olff et al., 2005; Bronner et al., 2009). Continuous avoidance and ineffective, maladaptive coping can lead to persistence of intrusive thinking and negative emotions like fear, anxiety, and depression; thus, passive coping can contribute to both the onset and maintenance of stress-related psychiatric disease, as does cognitive inflexibility (Foa and Kozak, 1986; Wenzlaff et al., 1988; Creamer et al., 1992). Thus, although fear dysregulation is undoubtedly central to the symptomatology of PTSD, targeting the underlying cognitive dysfunction associated with mPFC dysregulation may improve treatment outcomes for PTSD patients.

Animal models of PTSD are limited in that they cannot recreate the uniquely human experience of the disorder in total. Animal models can, however, effectively model defined dimensional components of behavior that resemble specific symptom clusters, allowing researchers to pursue mechanistic questions addressing the neurobiological circuits underlying the dysregulation of those behavioral dimensions. This is supported by neuroimaging studies that characterize specific neural circuits that are dysregulated in PTSD patients (Bremner et al., 1995, 1997; Liberzon et al., 1999; Rauch et al., 2006; Bryant et al., 2008). Further, the advent of sophisticated preclinical chemogenetic and optogenetic tools to selectively manipulate fear-related circuitry and plasticity can advance efforts to elucidate the neurobiological mechanisms of behavioral therapy for the treatment of PTSD. This review will highlight preclinical findings from our lab using fear extinction, not as a dependent measure, but as a model of exposure therapy, with an emphasis on the effects of extinction in restoring cognitive flexibility and active coping behavior that has been compromised after chronic unpredictable stress (CUS).

## Modeling PTSD Impairments in Rodents Using Chronic Unpredictable Stress (CUS)

Cognitive flexibility is an executive function mediated by the mPFC that is impaired in patients with PTSD (Birrell and Brown, 2000; Walter et al., 2010; Olff et al., 2014). The attentional set shifting test (AST) measures cognitive flexibility performance in rodents, and the extradimensional (ED) set shifting stage of the AST relies specifically on the function of the mPFC (Birrell and Brown, 2000; Bissonette et al., 2008). Our lab has extensively used the CUS paradigm to model the hyperarousal and medial prefrontal dysfunction observed in PTSD. The CUS procedure entails a series of several varied and robust acute psychogenic stressors applied once daily for a period of 2 weeks (Bondi et al., 2008, 2010). It is important to note that, in addition to preclinical models utilizing chronic stress to induce PTSD symptoms, models utilizing acute stress [such as the single prolonged stress (SPS) model; Lisieski et al., 2018] can also induce distinct PTSD-like phenotypes in rodents (see Goswami et al., 2013). Indeed, according to the DSM-V, both repeated and acute exposure to trauma can lead to a PTSD diagnosis. Therefore, the use of both chronic and acute stress models in the study of PTSD is informative, since the etiology of PTSD is complex (Scott and Stradling, 1994; Cloitre et al., 2009).

We have shown that this CUS treatment impairs performance on the ED set shifting stage of the AST (Bondi et al., 2008, 2010). Similar to the mPFC hypoactivity observed in PTSD patients, CUS decreases mPFC responsivity to afferent input in rodents (Jett et al., 2017). Other chronic stress paradigms also negatively alter the excitability of mPFC pyramidal neurons, and mPFC-mediated behaviors (Liston et al., 2006; Yuen et al., 2012). SPS is another rodent model of PTSD that induced impairments in executive function, including set shifting (George et al., 2015). We have also shown that set shifting impairment induced by CUS is reversed by several chronic and acute pharmacological interventions (Bondi et al., 2008, 2010; Naegeli et al., 2013; Jett et al., 2015).

In addition to cognitive impairment, uncontrollable and unpredictable stress in rodents can induce passive coping behaviors, consistent with avoidance symptoms observed in PTSD (Whitaker et al., 2016). Coping behavior is modulated by the mPFC in a top-down manner. In rodents, behavioral coping strategy in response to a threatening stimulus can be evaluated using the shock probe defensive burying test (SPDB; Lapiz-Bluhm et al., 2008). The SPDB involves placing a rat in a cage filled with bedding, with an electrified probe at one end of the cage (Fucich and Morilak, 2018). The rat approaches the probe and receives a shock, which evokes a rise in norepinephrine concentration in the lateral septum (LS) and a rise in plasma ACTH, an indicator of perceived stress (Bondi et al., 2007). Rats then engage in active coping behavior, defined by the amount of time they spend burying the probe with bedding, an ethologically-relevant defensive response, or passive coping behavior, defined by the amount of time spent immobile. Coping behaviors are assessed by analyzing both of these measures independently, and the relative amount of active vs. passive coping can then be expressed as a ratio (Fucich and Morilak, 2018). Following shock-probe exposure, we showed that rats allowed to bury the probe showed a return to baseline ACTH levels faster than rats that were unable to bury the probe because the bedding had been removed (Bondi et al., 2007). Thus, active burying in response to shock-probe exposure is an effective coping strategy that decreases stress. Further, the mPFC modulates activity in the LS, which promotes active coping in the SPDB test (Treit et al., 1993; Shah et al., 2004; Bondi et al., 2007). We have shown that CUS produces a shift from active to passive coping in the shock probe test, (Jett et al., 2015), modeling the avoidance behaviors seen in PTSD, and pharmacological interventions such as ketamine, vortioxetine, and desipramine prevent and/or reverse the stress-induced shift to passive coping (Bondi et al., 2007; Jett et al., 2015; Hatherall et al., 2017). These studies have highlighted the modulatory influence of monoaminergic neurotransmitters such as norepinephrine and serotonin, the targets of drugs such as traditional reuptake blocking antidepressants, and the essential role of glutamate as the primary excitatory neurotransmitter mediating the function and plasticity of prefrontal cortical circuits.

In sum, CUS produces functional and behavioral deficits similar to the mPFC-related cognitive impairment and avoidance-related symptoms seen in PTSD patients. Therefore, we used CUS to evaluate the therapeutic capacity of our rodent model of exposure therapy in reversing these effects.

## Fear Extinction as a Preclinical Model of Exposure Therapy

Cognitive behavioral therapy (CBT), developed by psychiatrist Aaron Beck, targets the underlying cognitive dysfunction observed in patients with psychiatric disorders, rather than treating only the individual symptoms that stem from those cognitive biases and cognitive dysfunction (Beck, 1976). Psychotherapeutic treatments for PTSD attempt to modify an individual’s cognitive appraisal of their fear, and may also involve repeated exposure to fear-provoking stimuli (Foa et al., 1989). Cognitive behavioral therapies, of which exposure therapy is but one example, also aim to improve active adaptive coping (Brewin, 1996; Beck, 2005). Exposure-based therapies engage areas of the brain, such as the hippocampus, PFC and amygdala, that are affected by chronic stress and are associated with PTSD and related neuropsychiatric disorders (Mahan and Ressler, 2012). Indeed, individuals that responded to prolonged exposure treatment had greater baseline hippocampal volume than treatment non-responders (Rubin et al., 2016). Cognitive behavioral therapies, including exposure therapy, increase activation of the ventrolateral and dorsolateral PFC after treatment, and are effective in ameliorating PTSD symptoms (Helpman et al., 2016; Yang et al., 2018). Thus, effective cognitive behavioral therapies may restore compromised activity in the mPFC, a regulator of executive function and emotional modulation.

Fear extinction is a form of safety learning that consists of the formation of a new memory in the ventromedial PFC (vmPFC; Milad and Quirk, 2002). Cue-conditioned fear extinction consists of a decrease in fear response (i.e., freezing) that results from the repeated exposure to a conditioned fear stimulus (i.e., a tone), that is not reinforced or punished (Martinez et al., 2012; Milad and Quirk, 2012). Fear conditioning association occurs in the basolateral and central amygdala, which have reciprocal inhibitory connections to the infralimbic (IL) cortex in the vmPFC. This bears resemblance to the process of exposure therapy, whereby patients, by repeated exposure to fear-provoking stimuli learn that they are no longer threatening, and as a result suppress their fear behavior. Similar to the cognitive reappraisal during CBT, fear extinction requires cognitive flexibility, i.e., modifying a previously learned association based on feedback from the environment.

## Extinction Learning Reverses Stress-Induced Deficits in Set Shifting and Promotes Active Coping

The effects of CUS are, at least partly, due to the attenuation of glutamatergic activity in the mPFC (Jett et al., 2017). Chronic stress induces reductions in apical dendritic spine numbers and dendritic length in the mPFC (Liston et al., 2006; Holmes and Wellman, 2009). Chronic stress also reduces AMPA receptor and NMDA receptor-mediated synaptic transmission, decreases the expression of glutamate receptors in the mPFC, and alters the expression and phosphorylation status of signaling molecules that mediate the transduction of neurotrophic signaling pathways that promote synaptic plasticity (Trentani et al., 2002).

**Figure 1:**
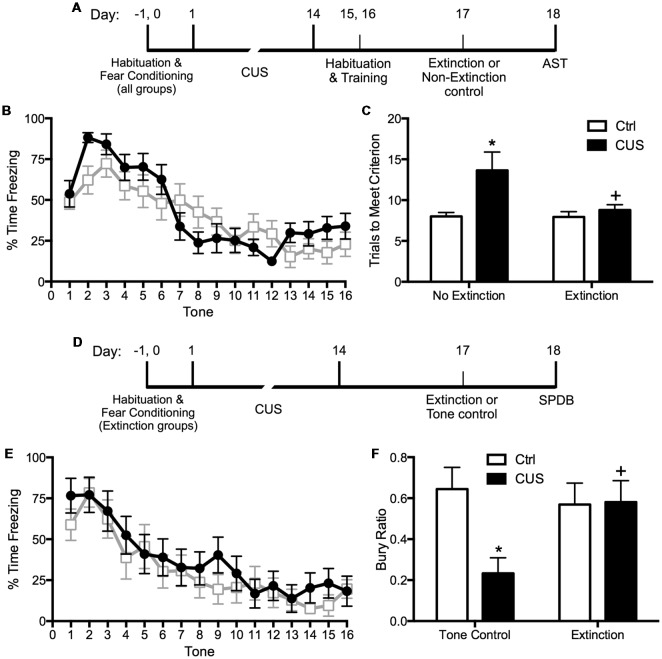
Effects of chronic unpredictable stress (CUS) and extinction therapy on attentional set-shifting and coping behavior on the shock-probe defensive burying test (SPDB). **(A)** Time line for the experiment testing the effects of chronic stress and extinction therapy on cognitive set-shifting. **(B)** Extinction, administered 24 h before testing on the Attentional Set-shifting Test (AST), was comparable in the two extinction treatment groups (CUS and unstressed control; area under the curves, *p* > 0.65); *n* = 14 per group. **(C)** CUS induced a significant increase in the number of trials required to meet criterion (TTC) of six consecutive correct responses on the set-shifting task (*p < 0.05, CUS compared to unstressed controls in the non-extinction groups). Extinction treatment reversed the effect of stress, restoring performance to unstressed control levels (^+^*p* < 0.05, extinction compared to non-extinction in the CUS groups); *n* = 14–15 per group. **(D)** Time line for the experiment testing the effects of chronic stress and extinction therapy on coping behavior. **(E)** Extinction, administered 24 h before testing on the shock probe test, was comparable in the two extinction treatment groups (CUS and unstressed control; area under the curves, *p* > 0.55); *n* = 12 per group. Extinction control groups exposed to tone presentation but not fear conditioned (“Tone controls”) showed low levels of freezing during tone presentation (not shown). **(F)** CUS induced a significant decrease in the Bury Ratio [calculated as bury time/(bury time + immobility time)]; *p < 0.05, CUS tone controls compared to unstressed tone controls). Extinction treatment reversed the effect of stress, restoring the Bury Ratio to unstressed control levels (^+^*p* < 0.05, CUS-extinction compared to CUS-tone controls); *n* = 11–12 per group. Data expressed as mean ± SEM. Reproduced and adapted with permission from Fucich et al. (2016).

Extinction is a learning process that promotes plasticity in the mPFC. Extinction learning activates the mPFC, much like exposure therapy in humans, and it enhances the excitability of glutamatergic pyramidal neurons in the vmPFC (Burgos-Robles et al., 2007). Therefore, we reasoned that restoring pyramidal cell function by engaging rats in a session of cognitive training by extinction learning would reverse the stress-induced deficits in set shifting. To test this, we first fear conditioned the rats by a standard procedure of four shock-tone pairings prior to stress, to avoid any effect of stress on the initial strength of fear learning. We then exposed them to 2 weeks of CUS or unstressed control treatment. Three days after the end of stress, we exposed them to a single session of 16 extinction trials with presentation of tones but no shock, and tested them on the set-shifting test 24 h after extinction. Another group of extinction controls were exposed to the same tone presentation, but without prior fear conditioning so that no learning took place during the session. We observed that extinction reversed the effects of CUS on set shifting, restoring performance back to non-stressed control levels (Figures 1A–C; Fucich et al., 2016). Extinction alone had no effect on set-shifting in unstressed rats, and exposure to tones alone without prior fear-conditioning did not improve set-shifting in stressed rats. Thus, training with a single session of cue-conditioned fear extinction had a therapeutic effect, reversing stress-induced deficits in cognitive set shifting.

We also investigated whether fear extinction could reverse the chronic stress-induced avoidance behavior modeled by a shift from active to passive coping on the SPDB test. We hypothesized that fear extinction, by engaging the mPFC and its modulatory influence on activity in its downstream target, the LS, would effectively restore active coping in stressed animals. The procedure and timing were as above. Active coping was measured by time spent burying the shock probe, and passive coping was measured by immobility. CUS induced a shift from active to passive coping on the SPDB test, and a single session of extinction 24 h before testing effectively restored active coping behavior back to unstressed control levels (Figures 1D–F; Fucich et al., 2016). Therefore, extinction as a model of exposure therapy ameliorated mPFC-dependent cognitive dysfunction and promoted active coping behavior that had been compromised by chronic stress.

## Mechanisms Underlying the Therapeutic Effects of Fear Extinction After Stress: Activity of Pyramidal Cells in the Infralimbic Cortex

Neuroimaging studies in clinical populations provide insight into the neural alterations that occur after effective psychotherapy. Studies show that activity of the vmPFC (corresponding to the IL mPFC in the rat brain) before CBT predicts symptom improvement (Ritchey et al., 2011). By contrast, hypoactivity in the mPFC is associated with increased symptom severity in major depressive disorder and PTSD (Shin et al., 2006). In addition, a recent study conducted in humans showed that stimulating the vmPFC with spatiotemporally focused transcranial magnetic stimulation (TMS) enhanced fear extinction learning, as measured by skin conductance responses (Raij et al., 2018). Fear extinction learning activates the mPFC, and its downstream targets in rodents (Sotres-Bayon et al., 2004). Further, retention of extinction memory requires the activity of pyramidal neurons in the vmPFC of rats, and stimulation of the vmPFC results in a decreased conditioned freezing response during fear extinction (Do-Monte et al., 2015). Thus, vmPFC activation may be necessary for the therapeutic effects of psychotherapy.

The IL and prelimbic (PL) sub-regions of the mPFC mediate opposing effects on fear expression behavior. Specifically, inactivating the PL impairs the expression of fear, but does not affect fear extinction memory. Conversely, inactivating the IL does not impair fear expression, but blocks fear extinction memory (Sierra-Mercado et al., 2011). Thus, we focused our attention in these studies on the IL cortex. We hypothesized that the activity specifically of glutamatergic pyramidal cells, the principle output neurons of the vmPFC, mediate the therapeutic effects of extinction learning on cognitive set-shifting and active coping behavior that have been compromised by CUS.

**Figure 2:**
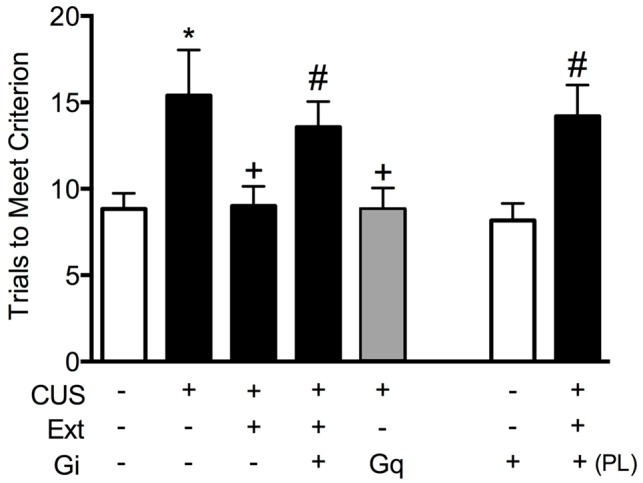
Activity of glutamatergic pyramidal cells in the infralimbic (IL) cortex is necessary and sufficient for the therapeutic effects of extinction on cognitive set-shifting compromised by chronic stress. CUS compromised cognitive flexibility, significantly increasing trials required to meet criterion (TTC) of six consecutive correct responses on the extradimensional (ED) set-shifting task (*p < 0.05, GFP/CUS/tone controls compared with GFP/unstressed/tone controls). Both extinction treatment and hM3Dq activation with clozapine-N-oxide (CNO) reversed the effect of stress, restoring TTC to unstressed control levels (^+^*p* < 0.05, GFP/CUS/extinction and hM3Dq/CUS compared with GFP/CUS/tone controls). hM4Di-mediated inhibition of IL projection neurons during extinction treatment prevented the therapeutic effect of extinction on set-shifting after CUS, as TTC were comparable to GFP/CUS/tone controls (^#^*p* < 0.05, hM4Di/CUS/extinction compared with GFP/CUS/extinction). Similarly, inhibiting glutamatergic neurons in prelimbic (PL) cortex during extinction also prevented the beneficial effect on set-shifting (^#^*p* < 0.05, hM4Di in PL/CUS/extinction compared with GFP/CUS/extinction). hM4Di-mediated inhibition of IL projection neurons alone in unstressed tone controls had no effect on set-shifting tested 24 h later; *n* = 5–9 per group. Data expressed as mean ± SEM. Reproduced and adapted with permission from Fucich et al. (2018).

To test the necessity of pyramidal cell activity in the vmPFC for the beneficial effects of extinction therapy on cognitive set shifting after stress, we used AAV viral-mediated delivery of an inhibitory Gi-coupled Designer Receptor Exclusively Activated by Designer Drug (DREADD) into the IL cortex, under the control of a CaMKIIα promoter to induce expression specifically in glutamatergic neurons. Controls received a microinjection of virus expressing an inert GFP construct. Four-to-five weeks total time was allowed for expression of the DREADD protein before testing. Thus, approximately 2 weeks after injection, rats began the CUS or unstressed control procedures. Three days after the end of stress, 30 min prior to the extinction therapy session, rats received an injection of the DREADD ligand clozapine-N-oxide (CNO, 1 mg/kg in 2% dimethylsulfoxide, i.p.) to selectively inhibit pyramidal cell activity in the IL cortex during extinction training. Rats were then tested for set-shifting on the AST 24 h after extinction. Our results showed that inhibiting pyramidal cell activity in the vmPFC, which had no effect on extinction itself, blocked the therapeutic effects of extinction on cognitive set shifting in stressed animals tested 24 h later (Figure 2; Fucich et al., 2018). Thus, activity of IL cortical pyramidal cells during extinction is necessary for its therapeutic effects on set shifting. We also tested whether activating these cells was sufficient to reverse the detrimental effects of stress on set-shifting. Rats received bilateral viral delivery of an excitatory Gq-coupled DREADD into the IL cortex. Three days after the end of CUS treatment, animals received an injection of CNO (1 mg/kg, i.p.) instead of extinction training, and were tested on AST 24 h post-injection. We found that transiently activating pyramidal cells in the IL cortex after CUS was sufficient to reverse the effects of stress on set shifting, mimicking the effects of extinction therapy (Figure 2; Fucich et al., 2018).

**Figure 3:**
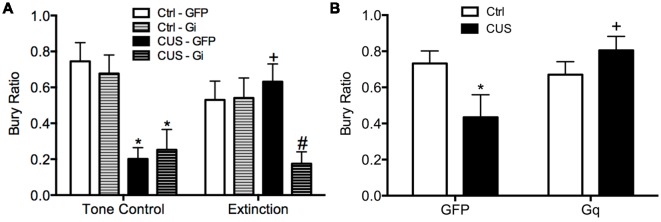
Activity of glutamatergic pyramidal cells in the IL cortex is necessary and sufficient for the therapeutic effects of extinction on the shift from active to passive coping behavior induced by chronic stress on the SPDB test. **(A)** CUS induced a significant decrease in bury ratio (*p < 0.01, for both GFP/CUS/tone controls and hM4Di/CUS/tone controls compared with GFP/unstressed/tone controls). Extinction treatment reversed the effect of stress, restoring the bury ratio to unstressed control levels (^+^*p* < 0.05, GFP/CUS/extinction compared with GFP/CUS/tone controls). Inhibition of glutamatergic neurons in IL during extinction prevented the therapeutic rescue of CUS-compromised bury ratio (^#^*p* < 0.02, hM4Di/CUS/extinction compared with GFP/CUS/extinction); *n* = 9–14 per group. **(B)** CUS induced a significant decrease in the bury ratio (*p < 0.05, GFP/CUS compared with GFP/unstressed controls). Activation of glutamatergic neurons in IL after transfection with the excitatory hM3Dq Designer Receptor Exclusively Activated by Designer Drug (DREADD) reversed the effect of stress, restoring the bury ratio to unstressed control levels 24 h after CNO administration (^+^*p* < 0.02, GFP/CUS compared to hM3Dq/CUS); *n* = 6–10 per group. Data expressed as mean ± SEM. Reproduced and adapted with permission from Fucich et al. (2018).

Using a similar DREADD strategy, we also investigated whether the activity of IL pyramidal neurons during extinction is necessary and sufficient for the therapeutic effects of extinction on active coping behavior on the SPDB in stressed animals, mediated by the LS (Treit et al., 1993; Bondi et al., 2007). The mPFC provides excitatory input to the LS, which in turn is composed of mainly inhibitory neurons that make reciprocal contacts with other sub-cortical regions associated with stress and fear, such as the amygdala, hypothalamus, and bed nucleus of the stria terminalis (Sheehan et al., 2004). Activity of LS neurons is increased during open arm exploration on the elevated plus maze (Thomas et al., 2013). By contrast, chronic stress blunts acute stress responsivity of the LS (Martinez et al., 1998). Thus, we reasoned that activity of IL pyramidal cells during extinction may induce plasticity downstream in the LS of stressed animals, promoting a shift back to active coping. We found that silencing pyramidal cells at the time of extinction prevented its beneficial effects on active coping behavior in stressed animals (Figure 3A), and that transiently activating pyramidal cells in the vmPFC after stress mimicked the beneficial effects of extinction therapy on coping behavior (Figure 3B; Fucich et al., 2018).

## Mechanisms Underlying the Therapeutic Effects of Fear Extinction After Stress: Activity-Dependent Protein Synthesis

In considering extinction as a learning process, it has been shown that extinction memory consolidation and retention require protein synthesis in the mPFC. Santini et al. (2004) further showed that extinction increased c-Fos expression in the dorsomedial PFC and vmPFC, but not in the insular cortex, suggesting that extinction learning initiates *de novo* protein synthesis in the mPFC. Based on what is known about the mechanisms underlying extinction learning and memory, together with our results discussed above showing the necessity of activity in the mPFC, we hypothesized that the therapeutic behavioral effects of extinction following chronic stress exposure may also be exerted through a process involving activity-dependent protein synthesis in the mPFC, similar to therapeutic mechanisms proposed for rapid-acting antidepressants, such as ketamine (Li et al., 2010; Autry et al., 2011; Monteggia et al., 2013; Duman et al., 2016).

We first observed that extinction increased phosphorylation at the S240/244 site of ribosomal protein S6 in the mPFC, but only in stressed rats (Fucich et al., 2016), indicating changes in protein synthesis (Roux et al., 2007; Knight et al., 2012). Changes in S6 phosphorylation are associated with activation of the mammalian target of rapamycin (mTOR) signaling cascade; mTOR activates the translational regulator ribosomal protein S6 kinase 1 (S6K1), which in turn activates ribosomal protein S6 by phosphorylation at serine S240/244 (Roux et al., 2007). The mTOR-p70S6K pathway has been linked to protein synthesis and structural changes in the mPFC that underlie the therapeutic effects of novel rapid-acting antidepressants (Li et al., 2010; Dwyer et al., 2015; Thomas and Duman, 2017).

**Figure 4:**
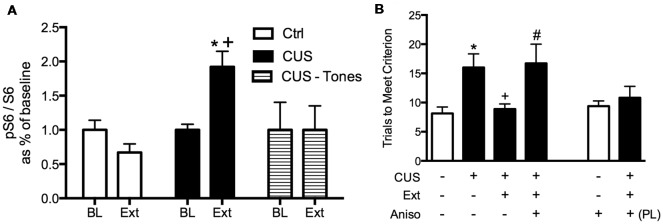
Therapeutic effects of extinction after CUS require protein synthesis in the IL medial prefrontal cortex (mPFC). **(A)** Extinction induced a significant increase in phosphorylation of ribosomal protein S6, reflecting initiation of *de novo* protein synthesis in the mPFC of CUS-treated rats (*p < 0.05, Extinction compared to Baseline), but not in unstressed controls, nor in CUS-tone control rats exposed to tone presentations but without prior fear conditioning (^+^*p* < 0.05, CUS Extinction compared to unstressed Extinction and to CUS tone controls); *n* = 4–6 per group. **(B)** Inhibition of protein synthesis by microinjection of anisomycin (50 μg/0.5 μl) into the IL cortex prior to extinction prevented the rescue of cognitive set-shifting that had been compromised by CUS. Chronic stress induced a significant increase in trials to criterion (TTC) on the set-shifting task (*p < 0.05, CUS-tone control-vehicle compared to unstressed-tone control-vehicle). Extinction treatment reversed the effect of stress, restoring TTC to unstressed control levels (^+^*p* < 0.05, CUS-extinction-vehicle compared to CUS-tone control-vehicle). Microinjection of anisomycin into IL cortex before extinction treatment prevented the beneficial effect of extinction on set-shifting compromised by CUS, as TTC were comparable to CUS tone controls (^#^*p* < 0.05, CUS-extinction-anisomycin compared to CUS-extinction-vehicle); *n* = 6–8 per group. Administering anisomycin into the IL cortex of unstressed animals had no effect on set-shifting. Similarly, as a site-specificity control, administering anisomycin into the PL cortex of stressed animals prior to extinction did not prevent the therapeutic effect of extinction. Data expressed as mean ± SEM. Reproduced and adapted with permission from Fucich et al. (2016).

We then tested whether *de novo* protein synthesis in the mPFC was necessary for the therapeutic behavioral effects of extinction on set shifting. Three days after the end of chronic stress treatment, animals received a local microinjection of the protein synthesis inhibitor, anisomycin into the mPFC 20 min prior to extinction. They were then tested on set-shifting 24 h later (Fucich et al., 2016). Blocking protein synthesis in the IL cortex during extinction had no effect on extinction learning itself. However, inhibiting protein synthesis in the IL during extinction completely blocked its subsequent therapeutic effects on set shifting (Figure 4). Importantly, anisomycin injection alone into the IL 24 h prior to testing did not affect set-shifting. Nor did blocking protein synthesis in the PrL cortex alter the therapeutic effects of extinction on set-shifting. Thus, these results support the hypothesis that activity-dependent protein synthesis specifically in the IL cortex is necessary for the therapeutic effects of extinction on cognitive set-shifting that has been compromised by chronic stress.

Our results show that protein synthesis in the IL is necessary for the therapeutic effects of extinction in stressed animals. We did not observe increased phosphorylation of ribosomal protein S6 after extinction in the mPFC of unstressed animals, consistent with previous reports suggesting that although protein synthesis is required for extinction, pS6 is not induced (Tedesco et al., 2014). However, we did observe phosphorylation of ribosomal protein S6 in the mPFC of stressed animals. Phosphorylation of S6 is not necessary for protein synthesis *per se*. However, induction of pS6 is associated with increased protein synthesis, and has been particularly associated with increased neural activity (Knight et al., 2012; Biever et al., 2015). Thus, the induction of pS6 in the mPFC of stressed animals but not in control animals suggests that a specific set of proteins may be translated uniquely after extinction in stressed animals that are not translated in control animals. Identification of these proteins, and their potential role in the plasticity underlying therapeutic effects of extinction as perhaps distinct from the plasticity underlying fear extinction memory, will require further investigation.

**Figure 5:**
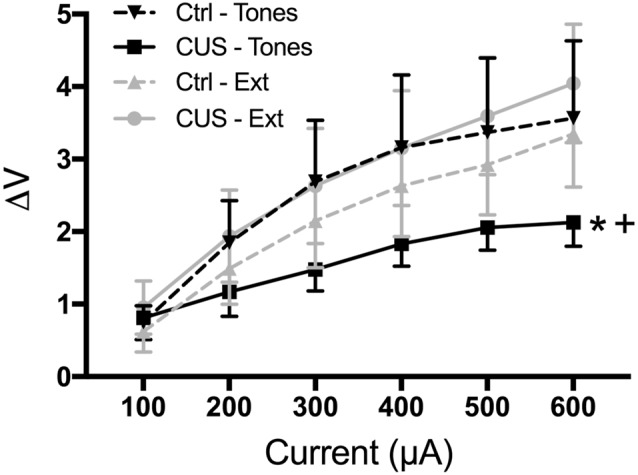
Extinction therapy rescues chronic stress-induced attenuation of afferent-evoked electrical responses in IL medial prefrontal cortex. CUS compromised afferent-evoked field potentials recorded in the mPFC in response to stimulation of the medial dorsal thalamus (*p < 0.01, CUS/tone controls compared with unstressed/tone controls). Extinction treatment reversed the effect of stress, restoring evoked responses to unstressed control levels (^+^*p* < 0.001, CUS/extinction compared with CUS/tone controls); *n* = 5–8 rats per group. Data expressed as mean ± SEM. Reproduced with permission from Fucich et al. (2018).

Thus, our results suggest that extinction restores cognitive, behavioral, and functional properties of the mPFC that are compromised by CUS and that resemble pathological changes in PTSD. However, it is not yet clear whether extinction initiates processes that reverse the aberrant maladaptive changes caused by stress in the mPFC, or if extinction learning instead initiates adaptive processes that can compensate for, but are distinct from, the stress-induced pathology in the mPFC. For example, chronic stress results in dendritic atrophy and reduced excitability of pyramidal cells in the mPFC, as well as reduced population responsivity to afferent input from the medial dorsal thalamus (Liston et al., 2006; Yuen et al., 2012; Jett et al., 2017). Such morphological and electrophysiological alterations are associated with impaired cognitive performance on mPFC-dependent tasks. We performed electrophysiological recordings suggesting that extinction learning restored afferent-evoked responses in the mPFC that had been compromised by chronic stress (Figure 5; Fucich et al., 2018). However, chronic stress also has been reported to increase GABA-mediated inhibition of IL pyramidal neurons (McKlveen et al., 2016). Thus, it is possible that extinction can directly activate pyramidal cells without necessarily altering aberrant GABAergic inhibitory activity induced by chronic stress.

## Summary and Future Directions

We and many others in both the basic and clinical literature have recognized that the fundamental process of exposure therapy is in fact a process of extinction (McNally, 2007; Hofmann, 2008; Craske et al., 2014). A question then is whether the mere extinction of a conditioned fear memory represents the entirety of the therapeutic effect of exposure therapy. We suggest that it does not. PTSD is more complex than just the memory of a stressful event, although that is an important and necessary component of a PTSD diagnosis. A related question might be whether cue-conditioned fear in and of itself represents a valid “model” of PTSD, which has been proposed (see Parsons and Ressler, 2013). We also suggest that it does not. First, it is unlikely that a few pairings of an innocuous tone with a 0.5 s, 0.7 mA foot shock is a traumatic stress. This was confirmed in our studies, in which fear conditioning alone had no effect on set-shifting (Fucich et al., 2016). Further, learning that the tone is to be feared in that context is not pathological. More practically, it would be circular logic to use cue-conditioned fear as a model of PTSD to test the extinction of cue-conditioned fear as a model of PTSD therapy. PTSD has other symptom domains, including a deficit of cognitive flexibility (Olff et al., 2014), which may contribute to the persistent and intrusive fear memory, but extends beyond fear memory alone. It is detected by neuropsychological tests that are not fear based, and from which the AST we use was back-translated (Birrell and Brown, 2000; Garner et al., 2006). PTSD also includes avoidance behavior and maladaptive coping, modeled by the shock probe test. Exposure therapy extinguishes the primary conditioned fear memory for the index event that initiated the pathology. But given the range of symptom domains in PTSD, we would argue that extinguishing the primary fear memory is not the sole therapeutic outcome of exposure therapy. Rather, we suggest that the process of learning involved in extinction induces plasticity in the mPFC that resolves or compensates for the pathology caused by the traumatic stress, which then accounts for the resolution of other symptom domains of PTSD. To test this in a preclinical model, and to avoid the circularity above, it is necessary to distinguish the target of the learning process *per se* (i.e., the cue-conditioned fear memory) from the dependent measures that characterize the stress-induced pathology and are used to assess therapeutic effect. A richer stress model than cue-conditioned fear alone is necessary to capture these other domains, hence our use of CUS to induce changes in cognition and coping behavior, measured by the set-shifting test and the shock probe test. To be clear, however, this is only necessary for a rigorous and valid preclinical test of the hypothesis that extinction learning, as a model of exposure therapy, induces plasticity in the mPFC that is therapeutic across symptom domains. It is not meant to imply that to treat PTSD it is necessary to induce a “second” fear memory that is then extinguished by exposure therapy to mitigate the pathology induced by the initial traumatic event. Extinction of the memory of the index event is the therapeutic learning process that accomplishes that. From a different perspective, this would also suggest that any process that induces similar plasticity in the mPFC would be similarly beneficial across PTSD symptom domains. This may be one mechanism by which ketamine has been reported to be effective in PTSD (Girgenti et al., 2017). More interestingly, this may explain the efficacy of other forms of CBT, the goal of which is not necessarily to extinguish memory of the traumatic event, but to train patients to utilize more flexible thinking and to disengage from automatic, reflexive, habitual responding with a perseverative negative bias (Gallagher and Resick, 2012). Further, it is important to recognize that the extinction of conditioned fear responses can be malleable, and subject to spontaneous recovery of fear (Myers and Davis, 2007), which may reflect preclinical correlates of re-experiencing or relapse. Thus, future work is needed to investigate the relationship between the reappearance of conditioned fear responses and the duration and stability of the therapeutic effects of extinction on other measures after stress.

Another consideration for future studies is gender. PTSD affects both men and women, with women being twice as likely to develop the disorder after a trauma (Haskell et al., 2010). The studies described in this review have included only male rats to date. In future work, we will include both sexes in our studies and monitor the estrous cycle at the time of testing, since estrous cycle stage can influence extinction learning as well as responses to stress (Viau and Meaney, 1991; Milad et al., 2009).

In addition to the mPFC, both chronic stress and fear extinction involve other brain regions relevant to PTSD symptomatology, such as the hippocampus and amygdala (Shin et al., 2006; Milad et al., 2007; Garcia et al., 2008; Mahan and Ressler, 2012). To date, our studies have only addressed the necessity of activity-dependent plasticity in the mPFC for the therapeutic effects of extinction in stressed animals. However, we have not yet investigated the possibility that plasticity in the mPFC during extinction is driven by activity in other brain regions that are also engaged by extinction learning. Indeed, we have reported that inhibiting the activity of pyramidal cells in the PrL cortex during extinction also blocked the therapeutic effects of extinction on set shifting. By contrast, and unlike IL, inhibiting protein synthesis in the PrL was not sufficient to block the therapeutic effects of extinction on set shifting. These results suggest that activity-dependent plasticity induced by extinction in the vmPFC interacts with activity in other components of the fear learning circuit to reverse cognitive impairments caused by stress. In this manner, extinction-induced plasticity in the vmPFC may enhance the function of downstream target circuits, for example by facilitating the inhibitory influence of PrL on the amygdala, or by reversing maladaptive plasticity in the hippocampal-PFC pathway caused by stress (Cerqueira et al., 2007; Koenigs and Grafman, 2009). Future work will be needed to investigate the circuit-level plasticity that may be necessary for the therapeutic effects of extinction in specific components of these extended vmPFC networks.

The fact that extinction induced phosphorylation of ribosomal protein S6 only in stressed animals suggests that the molecular machinery underlying plasticity (e.g., S6 induction) may be specifically dysregulated in the stressed brain, and that extinction initiates unique molecular processes related to protein synthesis and plasticity in the stressed brain. Thus, the observation that extinction requires activity-dependent protein synthesis in the mPFC for its therapeutic effects in stressed animals prompts two important questions for future investigation. The first is to ask what proteins are synthesized in the vmPFC that lead specifically to plasticity mediating the therapeutic effects of extinction, and whether they are distinct from factors responsible for the consolidation and retention of extinction memory *per se*. The second is to identify the upstream molecular factors and signaling pathways that initiate the protein synthesis mechanisms responsible for the therapeutic benefits of extinction. Several molecular pathways have been shown to be necessary for extinction memory consolidation, such as MAPK/Erk, PI3K/Akt, and BDNF (Hugues et al., 2004; Kritman and Maroun, 2013; Rosas-Vidal et al., 2014). Because these pathways mediate long-lasting plastic changes associated with extinction memory, they may also be involved in the lasting therapeutic effects of extinction. Indeed, several of these same signaling pathways have been implicated in the mechanisms of action of both traditional and novel rapid-acting antidepressant drugs (Autry et al., 2011; Thomas and Duman, 2017). Identification of upstream factors and signaling pathways that initiate extinction-mediated protein synthesis, and downstream factors and pathways that mediate the resulting plasticity underlying its beneficial effects, may lead to the discovery of novel therapeutic targets and strategies to enhance the beneficial effects of extinction, and by translational extension, enhance the therapeutic efficacy of CBT for PTSD. More generally, identifying substrates and molecular mechanisms by which effective therapeutic interventions, whether behavioral or pharmacological, exert their beneficial effects will hopefully lead to the future development of more effective treatments, including rational evidence-based adjunct strategies combining complementary behavioral and pharmacotherapeutic approaches.

## Author Contributions

DP wrote and edited the manuscript. DM provided critical feedback and edited the manuscript.

## Conflict of Interest Statement

The authors declare that the research was conducted in the absence of any commercial or financial relationships that could be construed as a potential conflict of interest.
